# Opportunities and challenges of mRNA technologies in development of dengue virus vaccine

**DOI:** 10.3389/fimmu.2025.1520968

**Published:** 2025-03-05

**Authors:** Xiaoyang Liu

**Affiliations:** Department of International Health, Bloomberg School of Public Health, Johns Hopkins University, Baltimore, MD, United States

**Keywords:** dengue, ADE, mRNA vaccine, DENV vaccine, tropical disease, vaccine development

## Abstract

Dengue virus (DENV) is a mosquito-borne virus with a significant human health concern. With 390 million infections annually and 96 million showing clinical symptoms, severe dengue can lead to life-threatening conditions like dengue hemorrhagic fever (DHF) and dengue shock syndrome (DSS). The only FDA-approved vaccine, Dengvaxia, has limitations due to antibody-dependent enhancement (ADE), necessitating careful administration. The recent pre-approval of TAK-003 by WHO in 2024 highlights ongoing efforts to improve vaccine options. This review explores recent advancements in dengue vaccine development, emphasizing potential utility of mRNA-based vaccines. By examining current clinical trial data and innovations, we aim to identify promising strategies to address the limitations of existing vaccines and enhance global dengue prevention efforts.

## 1 Introduction

Dengue virus, a mosquito-borne flavivirus, presents a serious global health challenge, especially in tropical regions across South America, the Caribbean (including Puerto Rico), the Western Pacific, Eastern Mediterranean, Africa, and the Americas (1). The World Health Organization (WHO) estimates that around 390 million dengue infections occur annually, with approximately 96 million cases showing clinical symptoms. While many infections are asymptomatic, severe cases can be life-threatening. Dengue fever ranges from mild flu-like symptoms to severe forms such as dengue hemorrhagic fever (DHF) and dengue shock syndrome (DSS) (2). DHF is marked by increased vascular permeability, leading to plasma leakage, bleeding, and low platelet counts; if untreated, it can progress to DSS, characterized by severe hypotension and circulatory collapse requiring intensive medical support. From 2000 to 2019, reported dengue cases rose tenfold, largely due to the expanding reach of *Aedes aegypti* and *Aedes albopictus* mosquitoes, driven by climate change. This environmental shift has introduced dengue to regions previously considered dengue-free (3).

Currently, the only FDA-approved dengue vaccine is Dengvaxia (CYD-TDV), a live-attenuated, tetravalent vaccine developed by Sanofi Pasteur in 2015 to protect against all four dengue virus serotypes (DENV-1 to DENV-4) (4). Despite its significance, Dengvaxia has limitations, notably the risk of antibody-dependent enhancement (ADE). ADE occurs when non-neutralizing antibodies from previous infection or vaccination facilitate viral uptake and replication in subsequent infections, potentially worsening symptoms. This risk is particularly concerning for flavivirus-naïve individuals, especially young children, so Dengvaxia is restricted to individuals with confirmed prior dengue exposure (5). In May 2024, WHO prequalified a new vaccine candidate, TAK-003, which avoids inducing ADE but has shown a lower antibody response against DENV-3 compared to other strains, underscoring the need for a comprehensive and safe dengue vaccine (6). Furthermore, global vaccine coverage remains limited, with only a small percentage of the population vaccinated, primarily in endemic areas. This limitation highlights the urgent need for vaccine strategies that can provide broad protection without ADE risks.

Given the existence of four distinct DENV serotypes, a universal DENV vaccine must elicit strong, balanced immunity against all strains to prevent ADE. Recent research has made promising strides towards this goal- the creation of a DENV envelope vaccine using computationally optimized broadly reactive antigen (COBRA) algorithms. This candidate elicited broadly neutralizing antibodies against all four serotypes in both mice and rhesus macaques, regardless of prior DENV exposure (7). Additionally, another research on a tetravalent DENV virus-like particles (VLPs) vaccine reported that high levels of neutralizing antibodies against all four strains were induced in non-human primates with a one-year immunity longevity and no observance of ADE (8). Unfortunately, none of these candidates has moved to clinical trials. Further evaluation of these vaccine constructs’ safety and efficacy should be performed in clinical trials.

The success of mRNA technology in recent vaccines, notably COVID-19 vaccines, offers a promising platform for dengue vaccine development. mRNA vaccines are adaptable, cost-effective, and can be produced rapidly. They work by instructing cells to produce a harmless viral protein, which then stimulates the immune system to generate specific antibodies. As non-infectious vaccines, mRNA platforms provide a safe and potent immune response (9). This review examines recent advancements in dengue vaccine development, with a particular focus on mRNA vaccine candidates. By analyzing current clinical trial data and recent innovations, we aim to highlight promising strategies to overcome the limitations of existing vaccines and enhance global dengue prevention efforts.

## 2 Dengue epidemiology

The earliest documented dengue outbreak dates to 1779, with cases reported in Jakarta, Indonesia, and Egypt (10). In recent decades, dengue incidence has dramatically increased, putting nearly half the global population at risk. From 2000 to 2019, reported dengue cases surged from 505,430 to 0ver 5.2 million, with a corresponding rise in deaths from 960 to 4032 (11). Annual dengue infections are estimated at around 390 million, with 67-136 million cases exhibiting clinical symptoms, particularly in tropical and subtropical regions. In the United States, dengue fever incidence has historically been low, with recent spikes occurring between 2013 and 2016 (0.17-0.31 cases per 100,000) and peaking in 2019 (0.35 cases per 100,000). 94% of cases between 2010 and 2021 were travel related. Puerto Rico, however, has seen a higher incidence, with an average of 200 cases per 100,000 between 1980 and 2015, and almost all cases were locally acquired (12). The substantial increase in dengue infections is primarily attributed to the expanded distribution of its vectors, particularly *Aedes aegypti* and *Aedes albopictus* mosquitoes, driven by climate change. Rising temperatures, increased rainfall, flooding, and humidity extend mosquito breeding periods and reduce virus incubation times (13, 14). Additionally, social and environmental factors such as population density increase, population mobility, and inadequate water storage practices, are closely associated with dengue transmission, particularly in rural areas (15). A longitudinal study from 2007 to 2009 in Puerto Rico emphasized that water storage containers and discarded tries play critical roles in mosquito breeding, as most pupae were found in human-managed water containers, storage vessels, plant pots, and leaking water meters (16).

## 3 Clinically approved vaccines

### 3.1 Dengvaxia

Invented by Sanofi Pasteur, Dengvaxia (CYD-TDV) is a live-attenuated, tetravalent vaccine administered as a three-dose regimen. It was approved by the U.S. Food and Drug Administration (FDA) in 2019. Incorporating structural pre-membrane (prM) and envelope (E) genes of the four DENV strains with non-structural genes of the yellow fever 17D vaccine strain, Dengvaxia aims to provide tetravalent immunity to all strains of DENV by targeting the prM and E proteins (17). In individuals over 9 years old who had previously been infected with dengue, the vaccine efficacy (VE) achieved 91% (95%CI, 58-99%) and up to 93.2% efficacy against severe disease. However, in dengue-naïve children under 9, VE is around 45% (95%CI, -54-88%), limiting its suitability for this group (18, 19). Therefore, Dengvaxia works as a “fill-in” vaccine with patients’ prior exposure served as the initial prime. In a phase-II clinical trial (NCT00880893), the safety of Dengvaxia was evaluated in subjects aged 2 to 45 years in Singapore. Throughout the whole study, there were only three (0.3%) recorded serious adverse events (SAEs) in the vaccination group- acute leukemia of ambiguous lineage, tuberculosis lymphadenitis, and tension headache- and three cases of adverse events (AEs)- fever, rash, and cervical spondylosis (20). On the other hand, due to concerns about ADE, Dengvaxia is recommended only for individuals with prior dengue exposure.

### 3.2 TAK-003

TAK-003 (Takeda) is a live-attenuated, tetravalent vaccine, comprising of four DENV strains with the attenuated DENV serotype 2 strain (DENV-2) as the vaccine backbone and three other recombinant strains, swapping the prM and E genes of DENV-2 with DENV-1, DENV-3, or DENV-4 (**Table 1**) (21). Previous phase I and II clinical trials for TAK-003 vaccine had addressed its capability of eliciting tetravalent neutralizing antibody responses and polyfunctional T-cell responses. In a Phase III trial (NCT02747927), two doses administered to children aged 4 to 16 showed a vaccine efficacy (VE) of 66.2% (95% CI, 44.9-77.5) for seronegative and 76.1% (95% CI, 68.5-81.9) for seropositive recipients (22). Cumulatively, VE was 90.4% (95% CI, 82.6-94.7) and 85.9% (95% CI, 31.9-97.1) against hospitalizations related to dengue and DHF. After three years, VE declined to 54.3% (95% CI, 41.9-64.1) for virologically confirmed dengue (VCD) and 77.1% (95% CI, 58.6-87.3) against hospitalized VCD in initially seronegative participants. Efficacy remained stable in seropositive groups, with VE at 65% (95% CI, 58.9-70.1) against VCD and 86% (95% CI, 78.4-91.0) against hospitalized cases. In another phase III trial evaluating safety of TAK-003 (NCT03771963), among 168 participants, there were only five participants experienced SAEs with two subjects reporting moderate hepatic failure and severe osteoarthritis and three reporting bradycardia, inguinal hernia, and sepsis (21). However, no efficacy was observed against DENV-3 in seronegative individuals, and VE against DENV-2 declined over time, raising concerns about potential antibody-dependent enhancement (ADE) in these cases.

**Table 1:** Current licensed or trialed dengue vaccines.

Name	Valency	Formulation	Evaluation	Manufacturer	Efficacy	Adverse events	Dose Schedule	Year
**Dengvaxia**	Tetravalence	Chimeric combination of YFV/DENV1-4	Licensed	Sanofi Pasteur	The general VE^e^ against all four serotypes was 65.6%	ADE response occurred in dengue naïve individuals was the major safety concern	3	2015
**TAK-003**	Tetravalence	Chimeric viruses with DENV-2 PDK35 as the backbone	Pre-licensed on May 2024 by WHO	Takeda	The cumulative VE against DENV1-4 was 66.2%	The most common adverse events were injection site pain and headache	2	2006
**TV003/TV005**	Tetravalence	Genetic attenuated viruses	Phase-III clinical trial	NIAID^a^, Butantan, and Merck	Seroconversion rate^f^ for TV003 was 74% and 97% for TV005	Headache, rash, fatigue, and myalgia were the most common observed adverse events	1	2003
**TDEN F17/F19**	Tetravalence	Virus combination attenuated by PDK cells	Phase II clinical trial	WRAIR^b^ and GSK^c^	Seroconversion rate against DENV1-3 was 100% and 83.3% against DENV4	Arthralgia, fatigue, muscle aches, and pain behind eyes were observed in recipients	2	2017
**DPIV**	Tetravalence	Purified inactivated DENV1-4 with aluminum adjuvants	Phase I clinical trial	WRAIR and GSK	Tetravalent neutralizing antibodies were induced	There were few cases of moderate adverse events recorded during the trial	2	2012
**TVDV**	Tetravalence	DNA vaccine encoding prM and E proteins of DENV1-4 and adjuvanted with VAXFECTIN	Phase I clinical trial	WRAIR and U.S. NMRC^d^	Anti-DENV IFN-γ T cells response was stimulated	No severe adverse events were observed	3	2018
**V180**	Tetravalence	Recombinant prM and E proteins of DENV1-4 combined with multiple adjuvants	Phase I clinical trial	Merck & Co.	Seroconversion rate against all four serotypes was 85.7%	Injection site pain was the most common adverse event throughout the trial	3	2018

^a^National Institute of Allergy and Infectious Diseases; ^b^Walter Reed Army Institute of Research; ^c^GlaxoSmithKline; ^d^U.S. Naval Medical Research Center;

^e^VE refers to vaccine efficacy, which is measured by comparing the number of disease cases in the vaccinated group to that of the placebo group;

^f^Seroconversion rate is the percentage of individuals who develop detectable specific antibodies to a pathogen in their blood post vaccination or infection. This rate is a common indicator of vaccine effectiveness in immunological research.

## 4 Vaccines under evaluations in clinical trials

### 4.1 TV003/TV005

The National Institute of Allergy and Infectious Disease (NIAID) has spearheaded the development of a live-attenuated tetravalent dengue vaccine over the past 15 years, aiming to provide comprehensive protection against all four dengue virus serotypes while minimizing the risk of ADE. The initial candidate, TV003, utilized deletions of 30 and 31 nucleotides (Δ30 and Δ31) at the 3’ UTR of each serotype to create four monovalent strains- rDEN1Δ30, rDEN2/4Δ30, and rDEN3Δ30/3Δ31 (23). To achieve a more balanced infectivity, in the TV005 formulation, the doses of rDENV2/4Δ30 were increased 10 fold (24). From previous phase I trials (NCT01072786 and NCT01436422), comparing to TV003, TV005 demonstrated a higher and more stable frequency of seroconversion and stronger antibody response (**Table 1**). Both candidates conferred sterilizing immunity against DENV infection for at least 12 months with a booster dose at 6 months. Mild dengue-related rash was the most common AE, occurring in 66% (27/41) of TV003 recipients and 26%(37/144) of TV004 recipients, with occasional reports of fever and arthralgia (25). Following a second dose, antibody titers to all serotypes roughly doubled, confirming the establishment of sterilizing immunity. TV003/TV005 are now undergoing Phase III trials (NCT02406729) in dengue-endemic areas, led by Brazil’s Butantan Institute.

### 4.2 TDEN F17/F19

TDEN F17 vaccine is a tetravalent, live-attenuated vaccine that targets all four dengue virus serotypes. Derived from a natural viral isolate, it was attenuated through serial passages in primary dog kidney (PDK) cells. To enhance neutralizing antibody responses, the F17pre formulation adjusted attenuation levels by increasing PDK cell passages for DENV1 and reducing them for DENV4 (26). Clinical trial (NCT00350337) demonstrated that TDEN F17/F17Pre/F19 induced robust humoral responses with tetravalent response rates of 60%, 71.4%, and 66.7% after 2-dose administration. In a phase-II trial conducted in Puerto Rico (NCT00468858) achieved 100% seroconversion to tetravalent immunity in primed subjects (27, 28). In a pilot study on TDEN F17’s VE in children, a 52.6% seroconversion was achieved, suggesting the vaccine might be safe and effective in children. Administered in a two-dose regimen, TDEN F17 has been shown to be safe across ages 12 months to 50 years, though 31% of F17pre recipients exhibited viremia after the first dose, which was absent after the second (29). A five-year Phase I/II study in Thai children aged 6-7 (NCT00384670), followed by a third-dose booster trial one year later (NCT01843621), demonstrated strong long-term immunity: 100% seroconversion to DENV-1, -2, and -3 and 83.3% to DENV-4. No mortality or serious adverse events were reported, indicating the safety of TDEN F17 (30).

### 4.3 DPIV

Invented at Walter Reed Army Institute of Research (WRAIR), the tetravalent dengue purified inactivated vaccine (DPIV) is administered with two-dose schedule 28 days apart. The DENV-2 S16803 strain was chosen as the initial vaccine prototype, which was successfully propagated in Vero cells and tested safe and immunogenic in mice and rhesus monkeys with a 100% seroconversion after the second dose administration (31). Adjuvanted with AS01_E_(3-O-desacylcinomnophsphoryl lipid A) and AS03_B_(oil-in-water) by GlaxoSmithKline, the formalin-inactivated viruses from DENV-1 Westpac 74, DENV-2 S16803, DENV-3 CH53489, and DENV-4 TVP360 were incorporated into the tetravalent formulation of DPIV. In a phase-I trial conducted in Puerto Rico (NCT01702857), DPIV adjuvanted by AS01E/AS03B elicited neutralizing antibody responses against all four DENV serotypes in flavivirus-naïve adults, but the response wanned in 6 months after the second dose (32). In addition, the inactivated nature of DPIV poses a potential limit on immune responses to non-neutralizing epitopes on target envelope and capsid proteins. Viral challenge studies in rhesus macaques revealed the vaccine failed to control DENV infection and inadvertently led to antibody-dependent enhancement of DENV infection with increased levels of viremia, AST, IL-10, and IL-18 in challenged animals (33).

### 4.4 TVDV

The U.S. Army Medical Research and Material Command developed the tetravalent DNA vaccine (TBDV) against dengue, using a VAXFECTIN-adjuvanted VR1012 plasmid encoding the prM and E proteins from each DENV serotype. TBDV combines equal amounts of four monovalent plasmids derived from distinct strains: DENV1 (West Pacific 74), a modified DENV2 strain, and low-passage Philippine strains for DENV3 and DENV4 (34). In New Zealand white rabbits, TBDV induced seroconversion across all four serotypes. A Phase I clinical trial (NCT01502358) with 40 flavivirus-naïve participants demonstrated TBDV’s safety and its ability to elicit IFN-γ-producing T-cell responses without causing dengue-related rash or serious adverse events (SAEs) (34). The most common adverse events were mild, including fatigue (17/40), headache (18/40), and muscle aches (19/40). While neutralizing antibodies were not detected, T-cell responses were observed, with an average response rate of 66.3% across groups receiving either low-dose TBDV, low-dose TVDV adjuvanted with Vaxfectin, or high-dose TVDV adjuvanted with Vaxfection (34).

### 4.5 V180

Merck & Co. developed V180, a recombinant tetravalent dengue vaccine targeting DENV envelope and prM glycoproteins, administered in a three-dose regimen. In a Phase I randomized, placebo-controlled, double-blind study (NCT01477580), neither unadjuvanted nor aluminum-adjuvanted V180 formulations induced a strong immune response. However, six formulations with the ISCOMATRIX adjuvant achieved robust immunogenicity (GMT > 150) with seroconversion rates exceeding 85.7% for all DENV serotypes. Memory B cell responses for all serotypes were also observed in high-dose V180-ISCOMATRIX recipients, though increased adverse events, such as injection site pain and swelling, were noted (35). A subsequent Phase I trial (NCT02450838) evaluated V180, plain or adjuvanted with Alhydrogel, as booster in adults previously vaccinated with a live-attenuated tetravalent dengue vaccine. While V180 was well-tolerated and enhanced serum neutralization titers, it did not meet the predefined booster criteria (GMT > 150) for a positive immune response (36).

## 5 mRNA DENV vaccines

The remarkable success of COVID-19 mRNA vaccines has highlighted the potential for mRNA-based dengue (DENV) vaccines (**Table 2**). Unlike viral vectored vaccines, which carry risks of reversion to virulence and require complex culturing processes, mRNA vaccines are non-infectious and do not carry nucleic acids that can integrate into the host genome, eliminating the risks of mutagenesis and oncogenesis. Comparing to inactivated and protein subunit vaccines that usually require adjuvants to enhance immune responses, mRNA vaccines inherently stimulate both cellular and humoral immunity through antigen expression within host cells, as seen with COVID mRNA vaccines (37). Furthermore, comparing to other novel platforms, like VLPs vaccines that mimic the structure of viruses and rely on complex bioprocessing, mRNA vaccines’ cell-free manufacturing nature accelerates the production process and makes them highly scalable (9). Their modular and flexible designs not only allow for precise targeting of antigens but also ensure the adaptability to new serotypes, making them a versatile platform for global vaccine development (38).

**Table 2:** Summary of mRNA DENV vaccine candidates.

Name	Target Serotype(s)	Development Stage	Preliminary effect	Safety Data	Year
**Modified mRNA Vaccine**	DENV-1 (NS3, NS4B, NS5) ^c^	Preclinical (Mouse Model)	Strong CD8+ T-cell responses were elicited	Vaccine design reduced the risk of ADE ^f^ by avoiding inducing Nab ^g^	2019
**mRNA-LNP ^a^ Vaccine**	DENV-2 (prME, E80, NS1) ^d^	Preclinical (Mouse Model)	Sterilizing immunity was induced in immunocompetent mice; reduced spleen viral load was also observed	Heterologous ADE was observed with E80-mRNA vaccinated mice	2020
**DENV1 prM/E ^b^ mRNA-LNP Vaccine**	DENV-1 (prM, E)	Preclinical (Mouse Model)	Robust humoral and cellular responses were elicited; vaccinated immunocompromised mice were protected from lethal DENV challenge	No morbidity and mortality cases were observed among those vaccinated mice	2021
**Multi-target mRNA-LNP Vaccine**	All four serotypes (NS1, E-DIII) ^e^	Preclinical (Mouse Model)	Neutralizing antibody against all four serotypes was elicited along with strong T-cell responses	ADE potential was minimized with only 5% of cell enhancement detected ^h^	2022

^a^Lipid nanoparticle; ^b^Pre-membrane protein/Envelope protein; ^c^Modified mRNA vaccine encoded DENV-1’s non-structural epitopes from NS3, NS4B, and NS5; ^d^mRNA-LNP vaccine targeted structural proteins- prM/E and E80- and one non-structural protein, NS-1; ^e^Multi-target mRNA-LNP vaccine encoded NS-1 protein and envelope domain III to target all four serotypes; ^f^Antibody enhancement effect; ^g^Neutralizing antibody; ^h^Enhancement effect was measured by antibody-dependent analysis of DENV1-4 infection in K562 cells.

**Figure 1:**
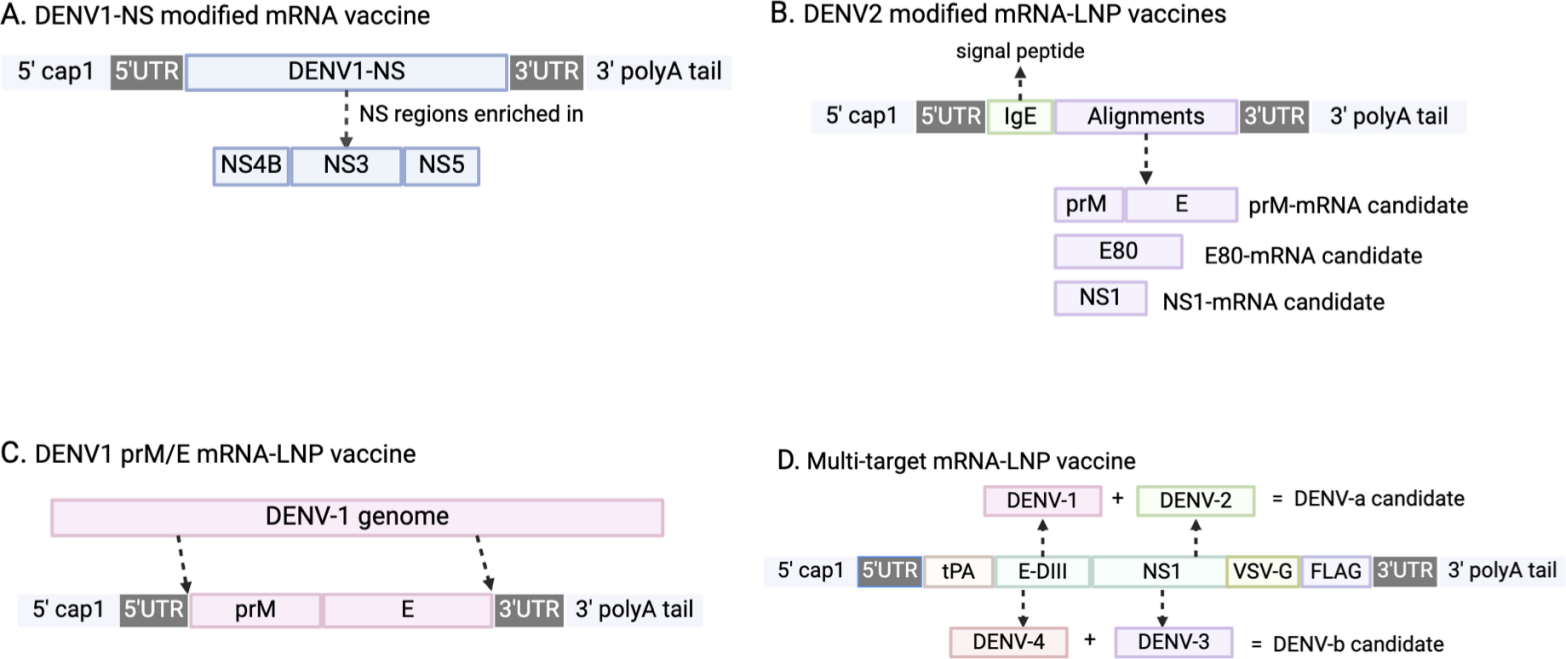
Schematic illustrations of mRNA DENV vaccine designs. **(A)** The major component of DENV1-NS modified mRNA vaccine is non-structural (NS) protein from DENV1 strain, and is enriched in NS4B, NS3, and NS5. **(B)** DENV2 modified mRNA-LNP vaccines incorporate a sequence of human IgE as a signal peptide to prompt alignments translocation. **(C)** The major component of this vaccine are sequences of pre-membrane(prM) and envelope (E) proteins extracted from DENV-1 genome. **(D)** The design incorporated tissue-type plasminogen activator (tPA) as the signal peptide, transmembrane and cytoplasmic domain of vesicular stomatitis virus G (VSV-G), and FLAG tag to aid the identification of target protein expression.

In 2019, Claude Roth and colleagues conducted a preclinical study in transgenic HLA Class-I (HLA-A0201, -A2402, B3501) mice to evaluate a modified mRNA vaccine against DENV-1 strain KDH0026A (**Figure 1A**) (39). This vaccine encoded immunodominant non-structural (NS) epitopes from NS3, NS4B, and NS5, designed to enhance CD4+ and CD8+ T-cell responses (40, 41). Encapsulated in lipid nanoparticles (LNPs) for delivery, the vaccine induced strong CD8+ T-cell responses, with 26% of CD8+ T-cells producing IFN-γ and TNF-α against HLA-B3501 peptide p49. The design intentionally avoided inducing neutralizing antibodies to minimize the risk of antibody-dependent enhancement (ADE) (39). However, the study did not explore responses to other DENV serotypes or test vaccine efficacy in a challenge model.

In 2020, another modified mRNA-LNP vaccine against DENV-2 strain 16681 was tested in mice (by Mengling Zhang et al), targeting two structural proteins, prME and E80, and one non-structural protein (NS1) (**Figure 1B**). Immunization generated high levels of DENV-2-specific IgG and strong T-cell responses, achieving sterilizing immunity against DENV-2 in immunocompetent BALB/c mice. E80-mRNA induced high titers of neutralizing antibodies (average PRNT50 titer of 13,000), while NS1-mRNA elicited a significant antibody response (PRNT50 titer of 12,000) and reduced viral loads in the spleen. However, NS1-mRNA alone did not induce neutralizing antibodies, limiting its utility as a primary vaccine component (42). Although E80-mRNA showed promise, it also induced high levels of heterologous ADE and cross-reactive immune responses, constraining its application.

In the following year, another similar mRNA-LNP vaccine against serotype 1, the DENV1 prM/E mRNA-LNP vaccine, was developed (by Clayton J. Wollner et al) (**Figure 1C**), targeting DENV-1 (strain 16007) with prM and E proteins with T7 promoter sequences and 5’/3’ URTs based on a ZIKV mRNA platform (43, 44). A two-dose regimen generated robust humoral and cellular responses, with antibody titers reaching 120,000 and neutralizing antibody titers of 420 measured by FRNT. This vaccine protected immunocompromised AG129 mice from lethal DENV-1 challenge without morbidity or mortality, indicating its potential effectiveness in immunocompromised populations (45). After vaccination, there was no morbidity or mortality signs shown among these mice, demonstrating a successful protection. Although it effectively elicited CD4+ and CD8+ T cell responses against DENV-1, it did not generate cross-reactive responses to other DENV serotypes.

Previous DENV mRNA vaccines only targeted one serotype; fortunately, in 2022, a multi-target mRNA-LNP vaccine formulation was designed and tested in mice (by Lihong He et al.) (**Figure 1D**) (46). The multivalent vaccines encoded NS1 and envelope domain III (E-DIII) to target all four DENV serotypes. The DENV-a candidate combined DENV-1 and DENV-2 antigens, while DENV-b combined DENV-3 and DENV-4. The efficacy of DENV-a, DENV-b, and DENV-ab was tested separately in mice. DENV-a elicited E-DIII specific IgG against DENV1 with an average antibody titer of 15,264 with a 20 μg dose. DENV-b immunized mice generated an E-DIII mean titer of 4608 in the 20 μg group. For NS-1, both DENV-a and DENV-b induced slightly higher titers than those of E-DIII, with mean titers of 61,440 and 32,768 (46). DENV-ab, a combination of DENV-a and DENV-b, produced the highest levels of neutralizing antibodies across all serotypes, and induced strong T-cell responses with high IFN-γ production. Additionally, ADE assays indicated minimal ADE potential (<5% of cells), underscoring the vaccine’s safety profile (46). The success of DENV-ab in mice suggests it as a promising candidate for further evaluation in larger animal models prior to clinical trials.

Comparing to traditional vaccine platforms, mRNA vaccines can mitigate ADE risks either through mutating fusion loop epitope of the E protein or by eliciting serotype-specific neutralizing antibodies. Previous research on DENV E protein has revealed three distinct domains, and domain II containing with an internal fusion loop was found to participate in membrane fusion and dimerization (47). By single point mutations of three fusion loop residues, tryptophan at position 101, leucine at position 107, and phenylalanine at position 108, the binding activity of cross-reactive anti-E antibodies, which were responsible for ADE, was greatly reduced (48). In addition, DENV1 prM/E mRNA-LNP vaccine did not induce cross-reactive antibodies that elicit heterotypic enhancement. In a focus reduction neutralization test (FRNT), DENV1 mRNA-vaccinated mice did not elicit neutralizing antibody against DENV2, and only a mild enhancement, around 1.2-fold, at 1/100 serum dilution was discovered (43). mRNA platform allows rapid development of vaccines, however global implementation of mRNA vaccines in dengue-endemic areas remains challenging. Vaccine stability is one of the greatest impediments, as mRNA formulations require ultra-cold storage, which is difficult to maintain in tropical climates with limited health infrastructures (49). Therefore, future efforts to advance LNP technology and thermostable mRNA formulations are essential to overcome this barrier.

Future research on DENV mRNA vaccines should focus on developing tetravalent vaccines that safely elicit both cellular and humoral immunity against all four serotypes. In addition to improving current LNPs formulation, lumazine synthase (LuS), can also be incorporated into future development of DENV mRNA vaccines (50). LuS oligomers, displaying as an efficient platform for antigen presentation, have already been successfully employed in other vaccine studies against infectious diseases, such as HIV, influenza, and rotavirus. When utilized as a scaffold, LuS was able to display spike glycoprotein from SARS-CoV-2 with decent yield and antigenicity in mice (51). Multimerization nature of LuS contributes to assembling antigens in a highly ordered fashion and promoting the activation of B cells receptors; thereby, potent and long-lasting immune responses in the germinal centers will be generated (52). When utilized as a protein cage, LuS carrying ovalbumin peptides OT-1 and OT-2 efficiently delivered and successfully stimulated Dendritic Cells (DCs) to produce OT-1 specific CD8+ T cells and OT-2 specific CD4+ T cells in mice (53). Immunity longevity of mRNA DENV vaccines is another aspect should be focused on. Though currently there is no published results on protection longevity, according to previous studies on SARS-CoV2 mRNA vaccine’s durability, it could infer that immunity provided by DENV mRNA vaccines might also last around one year (54). Moreover, further clinical studies and targeted research, which tailor dosing regimens, should be conducted to achieve consistent immunity in diverse populations, including children under five, the elderly, and immunodeficient people. The success of the DENV-1 prM/E mRNA-LNP vaccine in immunocompetent mice builds a promising foundation on the potential optimization of mRNA vaccines for immunocompromised population who has heightened risk of serve dengue. To further enhance efficacy, future strategies could include modifying LNP formulations to improve delivery, ensuring a controlled and sustained release of the mRNA payload. Additionally, incorporating adjuvants such as CpG oligonucleotides (55) or TLR7/8 (56) agonists may help boost innate and adaptive immune responses. Leveraging advanced antigen designs, such as self-assembling nanoparticles, could also ensure safety and efficacy in vulnerable populations. By addressing all aspects mentioned above, we will be one step closer to the successful deployment of mRNA vaccines in dengue-prevalent areas.

## 6 Conclusion

Currently, Dengvaxia is the only WHO-approved dengue vaccine, with global administration. However, in May 2024, WHO prequalified TAK-003, increasing global access to dengue vaccination. Among advanced candidates, TV003/TV005 has shown the strongest immune response in flavivirus-naïve recipients, with TV005 inducing fewer side effects like vaccine-related rash than TV003. TV003/TV005 provide 1-2 years of protection with a single dose, while Dengvaxia and TAK-003 offer protection for at least 6 and 4.5 years, respectively (57). Across all candidates, fewer than 1% of participants experienced serious adverse events, with rash as the most common side effect. Antibody-dependent enhancement (ADE) remains a concern, particularly for Dengvaxia, which poses an increased risk of severe dengue in seronegative individuals due to ADE. To reduce this risk, Dengvaxia is limited to those with prior dengue infection, confirmed by serology (58). Other vaccines show a lower ADE risk based on current trial data.

mRNA dengue vaccines offer significant advantages, including rapid development, scalability, and precise immune targeting Unlike live-attenuated vaccines, mRNA vaccines can be developed quickly without growing pathogens. mRNA vaccines can be engineered to encode antigens for all four dengue serotypes, ensuring balanced immune responses. Early preclinical trials indicate that mRNA vaccines effectively stimulate neutralizing antibodies and T-cell responses. Multi-target mRNA-LNP vaccines, targeting all four serotypes, show particular promise and may be advanced to clinical trials following safety and immunogenicity evaluations in non-human primates, such as rhesus macaques, cynomolgus macaques, or common marmosets (42). Once the candidate showed safety utilization in NHPs and was able to elicit strong immune responses, it could be advanced to clinical trials and eventually be brought to the market.

Future efforts in dengue vaccine development should focus on boosting efficacy and safety. Broadening immune responses could involve adding adjuvants like aluminum-based MF59 or AS01 to enhance Th2 responses in live-attenuated vaccines (59). Another promising approach involves specialized designs, such as self-assembling nanoparticles that mimic virus structure, enhancing antigen presentation and immune response. Nanoparticles can present multiple dengue antigens, while VLPs and multiepitope designs allow antigen presentation in a native conformation, increasing immune responses without viral replication risk (60–62). For mRNA vaccines, future work could refine lipid nanoparticle (LNP) formulations and nucleotide modifications, alongside novel antigen presentation platforms to optimize immune efficacy. Refining dosing regimens for diverse age groups, particularly under 4 and over 60 years old, will also be essential for broadening coverage and achieving herd immunity in dengue-endemic regions. It is pivotal to broaden the protection scope to achieve the goal of herd immunity in dengue-endemic areas.

In addition to scientific innovations, future directions in dengue vaccine development should also prioritize strategies for global accessibility. Overcoming logistical and economic barriers will require collaborative efforts, especially the international partnerships between local governments, non-governmental organizations (NGOs), and private industry to fund research, streamline regulatory approval, and subsidize vaccine costs in resource-limited settings. The success of TV003 highlights the potential of collaborative efforts in advancing dengue vaccine development. TV003 was developed by NIH but produced in partnership with a local company, Butantan Institute, in Brazil (63). This partnership not only facilitated the local production of TV003 but also underscored the importance of leveraging regional expertise to enhance vaccine accessibility and affordability in endemic areas. Such collaborations serve as a valuable model for advancing mRNA dengue vaccines, which could similarly benefit from partnerships with regional manufacturers to ensure scalability and cost-effectiveness. Innovative approaches like combining vaccination programs with mosquito control initiatives, such as Wolbachia-infected mosquito releases (64) or sterile insect techniques (65), could significantly enhance disease control efforts. To deepen understanding of dengue pathogenesis and vaccine-induced immunity, experimental methodologies like metabolomics and proteomics could identify biomarkers for vaccine efficacy, immune correlates of protection, and mechanisms of antibody-dependent enhancement. These insights would guide the optimization of vaccine designs and dosing regimens, ensuring more effective and accessible solutions for global dengue control.

## References

[1] Bhatt S Gething P Brady O Messina JP Farlow AW Moyes CL. The global distribution and burden of dengue. Nature. (2013) 496:504–7. doi: 10.1038/nature12060 PMC3651993 23563266

[2] Pintado Silva J Fernandez-Sesma A. Challenges on the development of a dengue vaccine: a comprehensive review of the state of the art. J Gen Virol. (2023) 104:1831. doi: 10.1099/jgv.0.001831 PMC10228381 36857199

[3] World Health Organization (WHO). Dengue and severe dengue(2024). Available online at: https://www.who.int/news-room/fact-sheets/detail/dengue-and-severe-dengue (Accessed May 26, 2024).

[4] Administration FaD. DENGVAXIA(2020). Available online at: www.fda.gov/vaccines-blood-biologics/dengvaxia (Accessed May 26, 2024).

[5] Teo A Tan HD Loy T Chia PY Chua CLL. Understanding antibody-dependent enhancement in dengue: Are afucosylated IgG1s a concern? PloS Pathog. (2023) 19:e1011223. doi: 10.1371/journal.ppat.1011223 36996026 PMC10062565

[6] Takeda. Takeda’s dengue vaccine candidate provides continued protection against dengue fever through 4.5 years in pivotal clinical trial(2022). Available online at: www.takeda.com/newsroom/newsreleases/2022/takedas-dengue-vaccine-candidate-provides-continued-protection-against-dengue-fever-through-4.5-years-in-pivotal-clinical-trial/ (Accessed May 26, 2024).

[7] Uno N Ross TM. Universal dengue vaccine elicits neutralizing antibodies against strains from all four dengue virus serotypes. J Virol. (2021) 95:e00658–20. doi: 10.1128/JVI.00658-20 PMC7851564 33208445

[8] Thoresen D Matsuda K Urakami A Ngwe Tun MM Nomura T Moi ML. A tetravalent dengue virus-like particle vaccine induces high levels of neutralizing antibodies and reduces dengue replication in non-human primates. J Virol. (2024) 98:e0023924. doi: 10.1128/jvi.00239-24 38647327 PMC11092354

[9] Pardi N Hogan MJ Porter FW Weissman D. mRNA vaccines - a new era in vaccinology. Nat Rev Drug Discovery. (2018) 17:261–79. doi: 10.1038/nrd.2017.243 PMC5906799 29326426

[10] Wu W Bai Z Zhou H Tu Z Fang M Tang B. Molecular epidemiology of dengue viruses in southern China from 1978 to 2006. Virol J. (2011) 8:322. doi: 10.1186/1743-422X-8-322 21703015 PMC3138434

[11] World Health Organization (WHO). Dengue: Global situation(2023). Available online at: https://www.who.int/emergencies/disease-outbreak-news/item/2023-DON498 (Accessed May 26, 2024).

[12] Chen LH Marti C Diaz Perez C Jackson BM Simon AM Lu M. Epidemiology and burden of dengue fever in the United States: a systematic review. J Travel Med. (2023) 30:taad127. doi: 10.1093/jtm/taad127 37792822

[13] Hawley WA. The biology of Aedes albopictus. J Am Mosq Control Assoc Supplement. (1988) 1:1–39. doi: 10.2149/tmh.2011-S04 3068349

[14] Higa Y. Dengue vectors and their spatial distribution. Trop Med Health. (2011) 39:17–27. doi: 10.2149/tmh.2011-S04 PMC3317606 22500133

[15] Schmidt WP Suzuki M Thiem VD White RG Tsuzuki A Yoshida LM. Population density, water supply, and the risk of dengue fever in Vietnam: cohort study and spatial analysis. PloS Med. (2011) 8:e1001082. doi: 10.1371/journal.pmed.1001082 21918642 PMC3168879

[16] Barrera R Amador M MacKay AJ. Population dynamics of Aedes aEgypti and dengue as influenced by weather and human behavior in San Juan, Puerto Rico. PloS Negl Trop Dis. (2011) 5:e1378. doi: 10.1371/journal.pntd.0001378 22206021 PMC3243685

[17] Guy B Briand O Lang J Saville M Jackson N. Development of the Sanofi Pasteur tetravalent dengue vaccine: One more step forward. Vaccine. (2015) 33:7100–11. doi: 10.1016/j.vaccine.2015.09.108 26475445

[18] Paz-Bailey G Adams L Wong JM Poehling KA Chen WH McNally V. Dengue Vaccine: Recommendations of the Advisory Committee on Immunization Practices, United States, 2021. MMWR. Recommendations and reports: Morbidity and mortality weekly report. Recommendations Rep. (2021) 70:1–16. doi: 10.15585/mmwr.rr7006a1 PMC8694708 34978547

[19] Hadinegoro SR Arredondo-García JL Capeding MR Deseda C Chotpitayasunondh T Dietze R. Efficacy and long-term safety of a dengue vaccine in regions of endemic disease. New Engl J Med. (2015) 373:1195–206. doi: 10.1056/NEJMoa1506223 26214039

[20] Capeding RZ Luna IA Bomasang E Lupisan S Lang J Forrat R. Live-attenuated, tetravalent dengue vaccine in children, adolescents and adults in a dengue endemic country: randomized controlled phase I trial in the Philippines. Vaccine. (2011) 29:3863–72. doi: 10.1016/j.vaccine.2011.03.057 21477675

[21] Patel SS Winkle P Faccin A Nordio F LeFevre I Tsoukas CG. An open-label, Phase 3 trial of TAK-003, a live attenuated dengue tetravalent vaccine, in healthy US adults: immunogenicity and safety when administered during the second half of a 24-month shelf-life. Hum Vaccines Immunother. (2023) 19:2254964. doi: 10.1080/21645515.2023.2254964 PMC10583633 37846724

[22] Rivera L Biswal S Sáez-Llorens X Reynales H López-Medina E Borja-Tabora C. Three-year efficacy and safety of Takeda’s dengue vaccine candidate (TAK-003). Clin Infect Dis: an Off Publ Infect Dis Soc America. (2022) 75:107–17. doi: 10.1093/cid/ciab864 PMC9402653 34606595

[23] Pintado Silva J Fenutria R Bernal-Rubio D Sanchez-Martin I Hunziker A Chebishev E. The dengue virus 4 component of NIAID’s tetravalent TV003 vaccine drives its innate immune signature. Exp Biol Med (Maywood NJ). (2022) 247:2201–12. doi: 10.1177/15353702231151241 PMC9899989 36734144

[24] Durbin AP. Historical discourse on the development of the live attenuated tetravalent dengue vaccine candidate TV003/TV005. Curr Opin Virol. (2020) 43:79–87. doi: 10.1016/j.coviro.2020.09.005 33164790 PMC7685199

[25] Whitehead SS Durbin AP Pierce KK Elwood D McElvany BD Fraser EA. In a randomized trial, the live attenuated tetravalent dengue vaccine TV003 is well-tolerated and highly immunogenic in subjects with flavivirus exposure prior to vaccination. PloS Negl Trop Dis. (2017) 11:e0005584. doi: 10.1371/journal.pntd.0005584 28481883 PMC5436874

[26] Sun W Cunningham D Wasserman SS Perry J Putnak JR Eckels KH. Phase 2 clinical trial of three formulations of tetravalent live-attenuated dengue vaccine in flavivirus-naïve adults. Hum Vaccines. (2009) 5:33–40. doi: 10.4161/hv.5.1.6348 18670195

[27] Thomas SJ Eckels KH Carletti I de la Barrera R Dessy F Fernandez S. A phase II, randomized, safety and immunogenicity study of a re-derived, live-attenuated dengue virus vaccine in healthy adults. Am J Trop Med Hyg. (2013) 88:73–88. doi: 10.4269/ajtmh.2012.12-0361 23208878 PMC3541749

[28] Bauer K Esquilin IO Cornier AS Thomas SJ Quintero Del Rio AI Bertran-Pasarell J. A phase II, randomized, safety and immunogenicity trial of a re-derived, live-attenuated dengue virus vaccine in healthy children and adults living in Puerto Rico. Am J Trop Med Hyg. (2015) 93:441–53. doi: 10.4269/ajtmh.14-0625 PMC4559678 26175027

[29] Watanaveeradej V Gibbons RV Simasathien S Nisalak A Jarman RG Kerdpanich A. Safety and immunogenicity of a rederived, live-attenuated dengue virus vaccine in healthy adults living in Thailand: a randomized trial. Am J Trop Med Hyg. (2014) 91:119–28. doi: 10.4269/ajtmh.13-0452 PMC4080550 24865677

[30] Simasathien S Thomas SJ Watanaveeradej V Nisalak A Barberousse C Innis BL. Safety and immunogenicity of a tetravalent live-attenuated dengue vaccine in flavivirus naive children. Am J Trop Med Hyg. (2008) 78:426–33. doi: 10.4269/ajtmh.2008.78.426 18337339

[31] Putnak R Barvir DA Burrous JM Dubois DR D’Andrea VM Hoke CH. Development of a purified, inactivated, dengue-2 virus vaccine prototype in Vero cells: immunogenicity and protection in mice and rhesus monkeys. J Infect Dis. (1996) 174:1176–84. doi: 10.1093/infdis/174.6.1176 8940206

[32] Diaz C Lin L Martinez LJ Eckels KH Campos M Jarman RG. Phase I randomized study of a tetravalent dengue purified inactivated vaccine in healthy adults from Puerto Rico. Am J Trop Med Hyg. (2018) 98:1435–43. doi: 10.4269/ajtmh.17-0627 PMC5953365 29512481

[33] Fernandez S Thomas SJ de la Barrera R Im-Erbsin R Jarman RG Baras B. An adjuvanted, tetravalent dengue virus purified inactivated vaccine candidate induces long-lasting and protective antibody responses against dengue challenge in rhesus macaques. Am J Trop Med Hyg. (2015) 92:698–708. doi: 10.4269/ajtmh.14-0268 25646261 PMC4385761

[34] Danko JR Kochel T Teneza-Mora N Luke TC Raviprakash K Sun P. Safety and immunogenicity of a tetravalent dengue DNA vaccine administered with a cationic lipid-based adjuvant in a phase 1 clinical trial. Am J Trop Med Hyg. (2018) 98:849–56. doi: 10.4269/ajtmh.17-0416 PMC5930886 29363446

[35] Manoff SB Sausser M Falk Russell A Martin J Radley D Hyatt D. Immunogenicity and safety of an investigational tetravalent recombinant subunit vaccine for dengue: results of a Phase I randomized clinical trial in flavivirus-naïve adults. Hum Vaccines Immunother. (2019) 15:2195–204. doi: 10.1080/21645515.2018.1546523 PMC6773383 30427741

[36] Durbin AP Pierce KK Kirkpatrick BD Grier P Sabundayo BP He H. Immunogenicity and safety of a tetravalent recombinant subunit dengue vaccine in adults previously vaccinated with a live attenuated tetravalent dengue vaccine: results of a phase-I randomized clinical trial. Am J Trop Med Hyg. (2020) 103:855–63. doi: 10.4269/ajtmh.20-0042 PMC7410446 32394880

[37] Gote V Bolla PK Kommineni N Butreddy A Nukala PK Palakurthi SS. A comprehensive review of mRNA vaccines. Int J Mol Sci. (2023) 24:2700. doi: 10.3390/ijms24032700 36769023 PMC9917162

[38] Rosa SS Prazeres DMF Azevedo AM Marques MPC. mRNA vaccines manufacturing: Challenges and bottlenecks. Vaccine. (2021) 39:2190–200. doi: 10.1016/j.vaccine.2021.03.038 PMC7987532 33771389

[39] Roth C Cantaert T Colas C Prot M Casadémont I Levillayer L. A modified mRNA vaccine targeting immunodominant NS epitopes protects against dengue virus infection in HLA class I transgenic mice. Front Immunol. (2019) 10:1424. doi: 10.3389/fimmu.2019.01424 31293584 PMC6598640

[40] Kurane I Zeng L Brinton MA Ennis FA. Definition of an epitope on NS3 recognized by human CD4+ cytotoxic T lymphocyte clones cross-reactive for dengue virus types 2, 3, and 4. Virology. (1998) 240:169–74. doi: 10.1006/viro.1997.8925 9454689

[41] Livingston PG Kurane I Dai LC Okamoto Y Lai CJ Men R. Dengue virus-specific, HLA-B35-restricted, human CD8+ cytotoxic T lymphocyte (CTL) clones. Recognition of NS3 amino acids 500 to 508 by CTL clones of two different serotype specificities. J Immunol (Baltimore Md: 1950). (1995) 154:1287–95. doi: 10.4049/jimmunol.154.3.1287 7529799

[42] Zhang M Sun J Li M Jin X. Modified mRNA-LNP Vaccines Confer Protection against Experimental DENV-2 Infection in Mice. Mol Ther Methods Clin Dev. (2020) 18:702–12. doi: 10.1016/j.omtm.2020.07.013 PMC7452130 32913878

[43] Wollner CJ Richner M Hassert MA Pinto AK Brien JD Richner JM. A dengue virus serotype 1 mRNA-LNP vaccine elicits protective immune responses. J Virol. (2021) 95:e02482–20. doi: 10.1128/JVI.02482-20 PMC8315947 33762420

[44] Richner JM Himansu S Dowd KA Butler SL Salazar V Fox JM. Modified mRNA Vaccines Protect against Zika Virus Infection. Cell. (2017) 169:176. doi: 10.1016/j.cell.2017.03.016 28340344

[45] Baldon LVR de Mendonça SF Ferreira FV Rezende FO Amadou SCG Leite THJF. AG129 mice as a comprehensive model for the experimental assessment of mosquito vector competence for arboviruses. Pathog (Basel Switzerland). (2022) 11:879. doi: 10.3390/pathogens11080879 PMC9412449 36015000

[46] He L Sun W Yang L Liu W Li J. A multiple-target mRNA-LNP vaccine induces protective immunity against experimental multi-serotype DENV in mice. Virol Sin. (2022) 37:746–57. doi: 10.1016/j.virs.2022.07.003 PMC9583182 35835315

[47] Modis Y Ogata S Clements D Harrison SC. A ligand-binding pocket in the dengue virus envelope glycoprotein. Proc Natl Acad Sci USA. (2003) 100:6986–91. doi: 10.1073/pnas.0832193100 PMC165817 12759475

[48] Lai CTsai WLin S Kao C Hu H King C Wu H Chang G. Antibodies to Envelope Glycoprotein of Dengue Virus during the Natural Course of Infection Are Predominantly Cross-Reactive and Recognize Epitopes Containing Highly Conserved Residues at the Fusion Loop of Domain II. Cell. (2008) 169(1):176. doi: 10.1128/jvi.00316-08 PMC2447043 18448542

[49] Uddin MN Roni MA. Challenges of storage and stability of mRNA-based COVID-19 vaccines. Vaccines. (2021) 9:1033. doi: 10.3390/vaccines9091033 34579270 PMC8473088

[50] Ladenstein R Morgunova E. Second career of a biosynthetic enzyme: Lumazine synthase as a virus-like nanoparticle in vaccine development. Biotechnol Rep (Amsterdam Netherlands). (2020) 27:e00494. doi: 10.1016/j.btre.2020.e00494 PMC7369331 32714852

[51] Zhang B Chao CW Tsybovsky Y Abiona OM Hutchinson GB Moliva JI. A platform incorporating trimeric antigens into self-assembling nanoparticles reveals SARS-CoV-2-spike nanoparticles to elicit substantially higher neutralizing responses than spike alone. Sci Rep. (2020) 10:18149. doi: 10.1038/s41598-020-74949-2 33097791 PMC7584627

[52] Roier S Mangala Prasad V McNeal MM Lee KK Petsch B Rauch S. mRNA-based VP8* nanoparticle vaccines against rotavirus are highly immunogenic in rodents. NPJ Vaccines. (2023) 8:190. doi: 10.1038/s41541-023-00790-z 38129390 PMC10739717

[53] Ra JS Shin HH Kang S Do Y. Lumazine synthase protein cage nanoparticles as antigen delivery nanoplatforms for dendritic cell-based vaccine development. Clin Exp Vaccine Res. (2014) 3:227–34. doi: 10.7774/cevr.2014.3.2.227 PMC4083076 25003097

[54] Figueroa AL Ali K Berman G Zhou H Deng W Xu W. Safety and durability of mRNA-1273-induced SARS-CoV-2 immune responses in adolescents: results from the phase 2/3 TeenCOVE trial. EClinicalMedicine. (2024) 74:102720. doi: 10.1016/j.eclinm.2024.102720 39091673 PMC11293523

[55] Klinman DM. Immunotherapeutic uses of CpG oligodeoxynucleotides. Nature reviews. Immunology. (2004) 4:249–58. doi: 10.1038/nri1329 15057783

[56] Dowling JK Mansell A. Toll-like receptors: the swiss army knife of immunity and vaccine development. Clin Trans Immunol. (2016) 5:e85. doi: 10.1038/cti.2016.22 PMC4910119 27350884

[57] Wilder-Smith A. Controlled human infection study underpins efficacy of the tetravalent live-attenuated dengue vaccine TV005. J Clin Invest. (2024) 134:e177610. doi: 10.1172/JCI177610 38299597 PMC10836794

[58] Center of Disease Control and Prevention (CDC). Dengue vaccination: What Everyone Should Know. Vaccines and Preventable Diseases. Available online at: https://www.cdc.gov/vaccines/vpd/dengue/index.html (Accessed June 1, 2024).

[59] Zhao T Cai Y Jiang Y He X Wei Y Yu Y. Vaccine adjuvants: mechanisms and platforms. Signal Transduct Target Ther. (2023) 8:283. doi: 10.1038/s41392-023-01557-7 37468460 PMC10356842

[60] López-Sagaseta J Malito E Rappuoli R Bottomley MJ. Self-assembling protein nanoparticles in the design of vaccines. Comput Struct Biotechnol J. (2015) 14:58–68. doi: 10.1016/j.csbj.2015.11.001 26862374 PMC4706605

[61] Mohsen MO Bachmann MF. Virus-like particle vaccinology, from bench to bedside. Cell Mol Immunol. (2022) 19:993–1011. doi: 10.1038/s41423-022-00897-8 35962190 PMC9371956

[62] Donaldson B Lateef Z Walker GF Young SL Ward VK. Virus-like particle vaccines: immunology and formulation for clinical translation. Expert Rev Vaccines. (2018) 17:833–49. doi: 10.1080/14760584.2018.1516552 PMC7103734 30173619

[63] Whitehead SS. Development of TV003/TV005, a single dose, highly immunogenic live attenuated dengue vaccine; what makes this vaccine different from the Sanofi-Pasteur CYD™ vaccine? Expert Rev Vaccines. (2016) 15:509–17. doi: 10.1586/14760584.2016.1115727 PMC4956407 26559731

[64] Pinto SB Riback TIS Sylvestre G Costa G Peixoto J Dias FBS. Effectiveness of Wolbachia-infected mosquito deployments in reducing the incidence of dengue and other Aedes-borne diseases in Niterói, Brazil: A quasi-experimental study. PloS Negl Trop Dis. (2021) 15:e0009556. doi: 10.1371/journal.pntd.0009556 34252106 PMC8297942

[65] Alphey L Benedict M Bellini R Clark GG Dame DA Service MW. Sterile-insect methods for control of mosquito-borne diseases: an analysis. Vector Borne Zoonotic Dis (Larchmont NY). (2010) 10:295–311. doi: 10.1089/vbz.2009.0014 PMC2946175 19725763

